# Characteristics of fall‐related head injury versus non‐head injury in the older adults

**DOI:** 10.1186/s12877-021-02139-4

**Published:** 2021-03-20

**Authors:** Sun Hyu Kim, Sunpyo Kim, Gyu Chong Cho, Ji Hwan Lee, Eun Jung Park, Duk Hee Lee

**Affiliations:** 1 Department of Emergency Medicine, University of Ulsan College of Medicine, Ulsan University Hospital, 877 Bangeojinsunhwando-ro, Dong-gu, 44033 Ulsan, Republic of Korea; 2 Department of Emergency Medicine, College of Medicine, Chosun University, Gwangju, Republic of Korea; 3 Department of Emergency Medicine, School of Medicine, Hallym University, Seoul, Republic of Korea; 4 Department of Emergency Medicine, Yonsei University College of Medicine, Seoul, Republic of Korea; 5 Department of Emergency Medicine, Ajou University School of Medicine, Suwon, Republic of Korea; 6 Department of Emergency Medicine, Ewha Womans University, Seoul, Republic of Korea

**Keywords:** Emergency department‐based Injury in‐depth Surveillance, Head injuries, Falls, Older adults

## Abstract

**Background:**

This study aimed to examine the characteristics of older adults patients who suffered a head injury after a ground-level fall in comparison to non-head injury patients as well as the factors associated with severity in those with head injury only.

**Methods:**

Patients were classified into two groups, the head injury group and the non-head injury group. The characteristics were compared and factors associated with head injury were evaluated. Factors relating to severe injury in the head injury group were also investigated.

**Results:**

The head injury group comprised 42 % of a study subjects. Male sex; fall time of 18:00–23:59; fall location of medical facility, transportation area, and public or commercial facility; fall in an outdoor area; fall during daily activity; alcohol ingestion; fall from stairs; non-slippery floor conditions; concrete flooring; sloped flooring; and presence of obstacles on the floor were risk factors for head injury in the older adults after a ground-level fall. Male sex and age over 70 years; fall time of 00:00–05:59; fall in a residential facility; fall in an indoor area; fall during daily activity; fall from stairs; non-slippery floor conditions; and presence of obstacles on the floor were factors associated with severe injury in the head injury group.

**Conclusions:**

Male sex with advanced age, indoor fall, and the presence of obstacles on the floor were risk factors for severe injury in the head injury group in older adults individuals who suffered a ground-level fall. It is necessary to develop appropriate ground-level fall prevention programs by evaluating the individual and environmental characteristics of older adults patients.

## Background

Ground-level falls are the most common cause of injury in the older adults [1]. Injuries associated with ground-level falls are becoming a serious global health problem as the older adults population grows [1, 2]. More than 60 % of traumatic brain injury (TBI) cases in older people are the result of falls, [3] and the prevalence of fall-related TBI has increased over the past few decades [3, 4]. Of all body regions that are injured in a fall, the head is most likely to be associated with mortality, [5, 6] and more than half of all fall-related deaths in older adults are related to head injuries [7]. TBI resulting from fall in the older adults is associated with declines in physical activity and cognitive function, as well as increasing healthcare cost with increasing hospital usage [3, 4].

Fall-related deaths are on the rise even though other trauma-related deaths are on the decline [8]. More than one-third of older adults patients who visit the emergency department (ED) after a fall-related injury revisit the ED or die within one year [9]. Therefore, it is important to reduce morbidity, mortality and economic losses by identifying the characteristics and risk factors for fall injury in the older adults.

Many studies have been performed on the characteristics of falls among the older adults, [2–7, 10–24] but no studies have analysed the characteristics of head injuries by comparing groups of head injury and non-head injury patients after falls in the older adults. This study aimed to examine the characteristics of older adults patients who suffered a head injury after a ground-level fall in comparison to non-head injury patients and the factors associated with severity in those with head injury only.

## Methods

This study looked at Emergency Department-based Injury In-depth Surveillance data from the Korea Center for Disease Control and Prevention Agency (KDCA), which has been collected since 2006. Data on older adults (aged 60 or older) who suffered a ground-level fall injury and presented to one of six university hospital EDs during an 8-year period from January 2011 to December 2018 were retrospectively reviewed. Because the fall injury rate is similar amongst those older than 65 years and 60–64 years of age, [22] older adults aged ≥ 60 years old were included in the study. The patients were divided into two groups, those who mainly suffered a head injury (head injury group) and those whose main injury involved another body part (non-head injury group). The study subjects included only those who had suffered a ground-level fall, not a fall from height. For each patient, the main injury was classified into seven regions according to the International Statistical Classification of Diseases and Related Health Problems 10th Revision (ICD-10): head and neck (S00-S19), thorax (S20-29), abdomen (S30-39), upper extremity (S40-69), hip and thigh (S70-79), lower extremity (S80-89) and multiple body region (T00-T14). Head and neck injury was defined as head injury since solitary neck injury is rare and many cases of neck injury are accompanied by head injury. This study was reviewed by the institutional IRB (UUH-IRB-2020-09-031) and consent was not required from the study subjects.

The general characteristics of the patients, including age, sex, season of injury occurrence, time of injury occurrence, location of injury occurrence, activity during injury occurrence, alcohol ingestion, education, occupation, mechanism of ground-level fall, environment of the floor when the fall occurred, blood pressure at the time of ED arrival, mode of transportation to the ED, state of consciousness, location of major injury, Excess Mortality Ratio-Adjusted Injury Severity Score (EMR-ISS), [14, 16, 25, 26] result of ED treatment, surgery, and injury severity were investigated. Age was divided into three groups, 60–69, 70–79, and 80 or older. Seasons were classified into spring (from March to May), summer (from June to August), autumn (from September to November) and winter (from November to February). The time of fall occurrence was divided into six-hour units. The location of fall occurrence was divided into six categories: residential facility, transportation area, medical facility, work place or sports facility, commercial or public facility and outdoor area; location was also classified as indoor or outdoor. The activities during fall occurrence were divided into four categories: paid work, unpaid work such as cleaning and cooking, leisure or sports activities, and daily activities such as using the toilet or showering. Education was categorized by highest level achieved, as follows: no education or elementary school, middle school, high school, and higher than college. The mechanism of ground-level fall was classified as fall from stairs, slip down on the same level, and others such as fall over. The floor condition was divided into slippery or not, and the type of floor was divided into concrete or other, such as soil or wood. The slope of the floor and whether an obstacle was present on the floor at the time of the fall were also investigated. The transportation to the ED was classified as public ambulance, individual transportation and transfer from another medical facility. Consciousness was divided according to AVPU scale: alert, verbal, pain, unresponsive. EMR-ISS is commonly utilized in South Korea as it is the injury severity scoring system based on the ICD-10. The excess mortality ratio for all ICD-10 codes from the Korean national injury database was used to grade the severity of every injury on a scale of 1–5. The EMR-ISS was calculated as the sum of squares of three maximum severity grades. The results of ED treatment were divided into discharge, admission to the general ward or intensive care unit, transfer to another facility, and death in the ED. The definition of a “severe” patient was one who required emergency surgery; was admitted to the intensive care unit (ICU) or transferred to other facility for further specialized care; or was died in the ED or dead on arrival. The head injury group was further sub-divided into a severe and non-severe injury group.

The Chi-square test and Student’s t-test were used to compare the characteristics between the head injury and non-head injury groups. Univariate logistic regression analysis was conducted on the patients’ general characteristics to identify the factors relating to head injury according to the patients’ individual and environmental characteristics at the time of injury. Multivariate logistic regression analysis was performed by selecting statistically significant variables (*p* < 0.05) in the univariate analysis. The education and occupation variables were not included in the logistic regression analysis due to a large number of missing values since the two were generally recorded only in hospitalized patients. Chi-square and Student’s t-tests were conducted to compare the general characteristics of the severe and non-severe head injury groups. To identify the factors associated with severe injury in the head injury group, univariate logistic regression analysis was also performed using the variables that were related to the general characteristics of the head injury group, and multivariate regression analysis was performed by selecting the statistically significant variables from the univariate analysis. IBM SPSS 24.0 (IBM Inc., Somers, NY, USA) was used for statistical analyses and *p* < 0.05 was defined as statistically significant.

## Results

A total of 34,366 patients were included in this study, with 14,433 (42.0 %) comprising the head injury group. The most common age group was 60–69 in the head injury group (40.1 %), whereas it was 70–79 in the non-head injury group (37 %) (*p* = 0.000). There were 7,795 (54.0 %) men in the head injury group and 5,747 (28.8 %) men in the non-head injury group (*p* = 0.000). The time of injury occurrence in the head injury group was most often 18:00–23:59, accounting for 34.1 % of patients, and 12:00–17:59 in the non-head injury group, with 34.7 % of patients. The two most frequent locations of injury occurrence were residential facilities (46.5 % vs. 56.9 %, head injury vs. non-head injury) and transportation areas (36.4 % vs. 26.6 %) in both groups (*p* = 0.000). Patients injured in an outdoor area accounted for 50.9 % of the head injury group and 41.3 % of the non-head injury group (*p* = 0.000). The patients were most often engaging in daily activities (76.2 % in the head injury group and 79.9 % in the non-head injury group) (*p* = 0.000). Twenty-three percent of patients were drinking alcohol at the time of injury in the head injury group, whereas the figure was 4.4 % in the non-head injury group (*p* = 0.000). The number of patients with a job was 16.7 % in the head injury group and 7.4 % in the non-head injury group (*p* = 0.000). Regarding the mechanism of fall, 15.5 % of the head injury group and 11.1 % of the non-head injury group fell from the stairs, and 42.7 % of the head injury group and 48.4 % of the non-head injury group slipped on the same level (*p* = 0.000). The floor was slippery in just 8.2 % of the cases in the head injury group and 11.9 % in the non-head injury group (*p* = 0.000). Fall on a concrete floor accounted for 84.2 % of the head injury group and 78.3 % of the non-head injury group (*p* = 0.000). The ground was sloped in 13.9 % of cases in the head injury group and 11.6 % in the non-head injury group (*p* = 0.000). There were obstacles on the floor 11.9 % of the time in the head injury group and 9.9 % in the non-head injury group (*p* = 0.000). Public ambulance (45.6 %) was the most common mode of transportation to the ED in the head injury group, while individual transportation (48.3 %) was more common in the non-head injury group (*p* = 0.000). In terms of consciousness at the ED, 92.9 % of the head injury group and 99.2 % of the non-head injury group were alert (*p* = 0.000). The most commonly injured region in the non-head injury group was the hip and thigh, accounting for 31.8 % of the patients. The EMR-ISS of the head injury group, 16.3, was higher than that of the non-head injury group, 11.3. The rate of admission to the general ward was 11.3 % in the head injury group and 42.0 % in the non-head injury group, while the rates of admission to the ICU were 8.0 % and 1.6 %, respectively. Surgery was performed on 11.1 % of patients in the head injury group and 26.6 % in the non-head injury group (*p* = 0.000), and severe patients accounted for 9.9 % of the head injury group and 3.8 % of the non-head injury group (*p* = 0.000) (Table 1).

**Table 1:** Characteristics of fall-related head injury and non-head injury in the older adults

	Head injury (*n* = 14,433)	Non-head injury (*n* = 19,933)	*p*
Average age, yrs	72.8.3 ± 8.6	74.5 ± 8.8	0.000
Age group, years old, (%)			0.000
60 ~ 69	5793 (40.1)	6457 (32.4)	
70 ~ 79	5263 (36.5)	7385 (37.0)	
≥ 80	3377 (23.4)	6091 (30.6)	
Sex, male (%)	7795 (54.0)	5747 (28.8)	0.000
Season of injury occurrence (%)			0.258
Spring (March ~ May)	3355 (23.2)	4487 (22.5)	
Summer(June ~ August)	3338 (23.1)	4603 (23.1)	
Autumn (September ~ November)	3963 (27.5)	5466 (27.4)	
Winter (December ~ February)	3777 (26.2)	5377 (27.4)	
Time of injury occurrence (%)			0.000
00:00–05:59	2273 (15.7)	3615 (18.1)	
06:00–11:59	2539 (17.6)	4528 (22.7)	
12:00–17:59	4704 (32.6)	6918 (34.7)	
18:00–23:59	4917 (34.1)	4872 (24.4)	
Location of injury occurrence (%)	*n* = 14,406	*n* = 19,895	0.000
Residential facility	6696 (46.5)	11,326 (56.9)	
Medical facility	359 (2.5)	556 (2.8)	
Sports facility or work place	309 (2.1)	498 (2.5)	
Transportation area	5238 (36.4)	5295 (26.6)	
Public or commercial facility	1361 (9.4)	1375 (6.9)	
Other outdoor area	443 (3.1)	845 (4.2)	
Indoor or outdoor fall injury (%)	*n* = 14,417	*n* = 19,922	0.000
Outdoor	7375 (50.9)	8228 (41.3)	
Activity during injury occurrence (%)	n = 14,170	n = 19,529	0.000
Paid work	291 (2.1)	439 (2.2)	
Unpaid work	534 (3.8)	709 (3.6)	
Sports or leisure activity	2547 (18.0)	2769 (14.2)	
Daily activity	10,798 (76.2)	15,612 (79.9)	
Alcohol ingestion, case n/total n (%)	2601/11,534 (22.6)	652/14,974 (4.4)	0.000
Education (%)	*n* = 1224	*n* = 2995	0.001
Uneducated or elementary school	570 (46.6)	1570 (52.4)	
Junior high school	214 (17.5)	499 (16.7)	
High school	270 (22.1)	614 (20.5)	
≥ College	170 (13.9)	312 (10.4)	
Occupation, employed or unemployed (%)	*n* = 3064	*n* = 9230	0.000
Employed	513 (16.7)	685 (7.4)	
Mechanism of fall (%)			0.000
Fall from stairs	2243 (15.5)	2220 (11.1)	
Slip down on same level	6157 (42.7)	9643 (48.4)	
Others including fall over	6033 (41.8)	8070 (40.5)	
Environment of floor while occur fall (%)			
Slippery condition of floor or non-slippery	*n* = 14,261	*n* = 19,590	0.000
Slippery	1165 (8.2)	2324 (11.9)	
Type of floor, concrete or others	*n* = 14,273	*n* = 19,582	0.000
Concrete	12,024 (84.2)	15,338 (78.3)	
Slope of floor, sloping or flat	*n* = 14,302	*n* = 19,646	0.000
Sloping	1981 (13.9)	2275 (11.6)	
Existing of obstacle, yes or none	*n* = 14,306	*n* = 19,649	0.000
Yes	1708 (11.9)	1905 (9.9)	
Blood pressure, mmHg	*n* = 12,000	*n* = 16,280	
Systolic blood pressure	142.1 ± 27.4	142.2 ± 25.3	0.813
Diastolic blood pressure	80.0 ± 14.5	79.2 ± 13.9	0.000
Transportation to ED (%)			0.000
Public ambulance	6588 (45.6)	7231 (36.3)	
Other medical facility	1377 (9.5)	3076 (15.4)	
Individual transportation	6468 (44.8)	9626 (48.3)	
Consciousness at ED (%)	*n* = 11,124	*n* = 15,546	0.000
Alert	10,331 (92.9)	15,424 (99.2)	
Verbal response	480 (4.3)	94 (0.6)	
Pain response	254 (2.3)	19 (0.1)	
Unresponsive	59 (0.5)	9 (0.1)	
Major injury region			
Head and neck	14,433 (100.0)		
Thorax		1712 (8.6)	
Abdomen		2556 (12.8)	
Upper extremity		4801 (24.1)	
Hip and thigh		6348 (31.8)	
Lower extremity		2624 (13.2)	
Multiple body region		1892 (9.5)	
EMR-ISS	16.3 ± 11.7 (n = 14,414)	11.3 ± 8.1 (n = 19,897)	0.000
Result of ED treatment (%)			
Discharge	11,196 (77.6)	10,813 (54.2)	0.000
Transfer to other facility	423 (2.9)	422 (2.1)	
Admission to general ward	1626 (11.3)	8362 (42.0)	
Admission to intensive care unit	1158 (8.0)	324 (1.6)	
Death at emergency department	30 (0.2)	12 (0.1)	
Operation, case n/total n (%)	1187/10,697 (11.1)	4064/15,287 (26.6)	0.000
Severe patients (%)	1425 (9.9)	748 (3.8)	0.000

*ED* emergency department; *EMR-ISS* Excess Mortality Ratio-adjusted Injury Severity Score

The main factors significantly associated with head injury after a ground-level fall in patients over 60 years of age were male sex (odds ratio 2.257, 95 % confidence interval 2.132–2.389, *p* = 0.000); time of injury, between 18:00 and 23:59 compared to 00:00 to 05:59 (1.167, 1.073–1.270, *p* = 0.000); injury location, medical facility (1.323, 1.061–1.651, *p* = 0.013), transportation area (1.147, 1.039–1.265, *p* = 0.006), or public or commercial facility (1.298, 1.160–1.452, *p* = 0.000) compared to residential facility; outdoor location (1.254, 1.146–1.372, *p* = 0.000); and unpaid work during injury occurrence compared to daily activity (1.234, 1.082–1.408, *p* = 0.0020). Alcohol ingestion (3.881, 3.488–4.318, *p* = 0.000); falling from stairs compared to falling over (1.476, 1.292–1.686, *p* = 0.000); non-slippery floor conditions (1.313, 1.202–1.433, *p* = 0.000); concrete flooring (1.178, 1.097–1.264, *p* = 0.000); sloped flooring (1.242, 1.105–1.396, *p* = 0.000); and presence of obstacles on the floor (1.120, 1.025–1.224, *p* = 0.013) were the other factors associated with head injury in older adults patients who suffered ground-level fall injuries (Table 2).

**Table 2:** Factors associated with fall-related head injury vs. non-head injury in the older adults

	Odds Ratio	95 % Confidence Interval	*P*
Univariate logistic regression analysis			
Age group, years old			
60 ~ 69	1.0		
70 ~ 79	0.794	0.756–0.835	0.000
≥ 80	0.618	0.585–0.653	0.000
Male, sex versus female	2.899	2.772–3.032	0.000
Season of injury occurrence			
Winter	1.0		
Spring	1.064	1.001–1.132	0.045
Summer	1.032	0.971–1.097	0.306
Autumn	1.032	0.974–1.094	0.288
Time of injury occurrence			
00:00–05:59	1.0		
06:00–11:59	0.892	0.830–0.958	0.002
12:00–17:59	1.081	1.014–1.153	0.017
18:00–23:59	1.605	1.503–1.714	0.000
Location of injury occurrence			
Residential facility	1.0		
Medical facility	1.092	0.953–1.251	0.204
Sports facility or work place	1.050	0.908–1.213	0.514
Transportation area	1.673	1.594–1.757	0.000
Public or commercial facility	1.674	1.544–1.815	0.000
Other outdoor area	0.887	0.787–0.999	0.048
Outdoor fall injury versus indoor fall injury	1.476	1.414–1.541	0.000
Activity during injury occurrence			
Daily activity	1.0		
Paid work	0.958	0.825–1.114	0.579
Unpaid work	1.089	0.971–1.222	0.146
Sports or leisure activity	1.330	1.254–1.411	0.000
Alcohol ingestion	6.396	5.846–6.997	0.000
Mechanism of fall			
Others including fall over	1.0		
Fall from stairs	1.352	1.263–1.446	0.000
Slip down on same level	0.854	0.816–0.894	0.000
Non-slippery, condition of floor versus slippery	1.513	1.405–1.629	0.000
Concrete, type of floor versus others	1.479	1.398–1.565	0.000
Sloping floor versus flat	1.228	1.151–1.309	0.000
Existing of obstacles on floor	1.231	1.149–1.318	0.000
Multivariate logistic regression analysis			
Male, sex versus female	2.257	2.132–2.389	0.000
Time of injury occurrence, versus 00:00–05:59			
06:00–11:59	0.876	0.800–0.960	0.004
18:00–23:59	1.167	1.073–1.270	0.000
Location of injury occurrence, versus residential facility			
Medical facility	1.323	1.061–1.651	0.013
Transportation area	1.147	1.039–1.265	0.006
Public or commercial facility	1.298	1.160–1.452	0.000
Outdoor fall injury versus indoor fall injury	1.254	1.146–1.372	0.000
Activity during injury occurrence, versus daily activity			
Paid work	0.693	0.561–0.856	0.001
Unpaid work	1.234	1.082–1.408	0.002
Sports or leisure activity	0.672	0.616–0.733	0.000
Alcohol ingestion	3.881	3.488–4.318	0.000
Fall from stairs, mechanism of fall versus others including fall over	1.476	1.292–1.686	0.000
Non-slippery, condition of floor versus slippery	1.313	1.202–1.433	0.000
Concrete, type of floor versus others	1.178	1.097–1.264	0.000
Sloping floor versus flat	1.242	1.105–1.396	0.000
Existing of obstacles on floor	1.120	1.025–1.224	0.013

The most common age group was 70–79 years (40.6 %) in the severe subgroup and 60–69 years (40.8 %) in the non-severe subgroup of the head injury group (*p* = 0.000). The proportion of males in the severe group was higher than that in the non-severe group (*p* = 0.000). The most common locations of injury were residential facilities and transportation areas in both groups. Ground-level fall injuries tended to occur in indoor areas more often in the severe group than the non-severe group (52.3 % vs. 48.7 %) (*p* = 0.010). Fifteen point five % of the severe group and 18.2 % of the non-severe group were engaged in a sports or leisure activity at the time of the injury (*p* = 0.016). Alcohol ingestion was more common in the non-severe group (*p* = 0.014) and employment status was not different between the two groups. Fall from stairs was the fall mechanism in 22.0 % of the severe group and 14.8 % of the non-severe group (*p* = 0.000). At the time of injury occurrence, the floor was sloped in 18.7 % of cases in the severe group and 13.3 % of those in the non-severe group (*p* = 0.000), and obstacles were present on the floor in 15.9 % of cases in the severe group and 11.5 % of those in the non-severe group (*p* = 0.000) (Table 3).

**Table 3:** General characteristics of fall-related head injury by severity in the older adults

	Severe (*n* = 1425)	Non-severe (*n* = 13,008)	*p*
Average age, yrs	73.7.3 ± 8.3	72.6 ± 8.6	0.000
Age group, years old, (%)			0.000
60 ~ 69	482 (33.8)	5311 (40.8)	
70 ~ 79	579 (40.6)	4684 (36.0)	
≥ 80	364 (25.5)	3013 (23.2)	
Sex, male (%)	874 (61.3)	6921 (53.2)	0.000
Season of injury occurrence (%)			0.702
Spring (March ~ May)	328 (23.0)	3027 (23.3)	
Summer(June ~ August)	333 (23.4)	3005 (23.1)	
Autumn (September ~ November)	376 (26.4)	3587 (27.6)	
Winter (December ~ February)	388 (27.2)	3389 (26.1)	
Time of injury occurrence (%)			0.015
00:00–05:59	251 (17.6)	2022 (15.5)	
06:00–11:59	261 (18.3)	2278 (17.5)	
12:00–17:59	479 (33.6)	4225 (32.5)	
18:00–23:59	434 (30.5)	4483 (34.5)	
Location of injury occurrence (%)	*n* = 1422	*n* = 12,984	0.000
Residential facility	761 (53.5)	5935 (45.7)	
Medical facility	38 (2.7)	321 (2.5)	
Sports facility or work place	36 (2.5)	273 (2.1)	
Transportation area	402 (28.3)	4836 (37.2)	
Public or commercial facility	139 (9.8)	1222 (9.4)	
Other outdoor area	46 (3.2)	397 (3.1)	
Indoor or outdoor fall injury (%)	*n* = 1423	*n* = 12,994	0.010
Outdoor	679 (47.7)	6666 (51.3)	
Activity during injury occurrence (%)	*n* = 1394	*n* = 12,776	0.016
Paid work	36 (2.6)	255 (2.0)	
Unpaid work	44 (3.2)	490 (3.8)	
Sports or leisure activity	216 (15.5)	2331 (18.2)	
Daily activity	1098 (78.8)	9700 (75.9)	
Alcohol ingestion, case n/total n (%)	224/1140 (19.6)	2377/10,394 (22.9)	0.014
Education (%)	*n* = 515	*n* = 709	0.001
Uneducated or elementary school	224 (43.5)	346 (48.8)	
Junior high school	100 (19.4)	114 (16.1)	
High school	118 (22.9)	152 (21.4)	
≥ College	73 (14.2)	97 (13.7)	
Occupation, employed or unemployed (%)	*n* = 1273	*n* = 1791	0.633
Employed	218 (17.1)	295 (16.5)	
Mechanism of fall (%)			0.000
Fall from stairs	313 (22.0)	1930 (14.8)	
Slip down on same level	635 (44.6)	5522 (42.5)	
Others including fall over	477 (33.5)	5556 (42.7)	
Environment of floor while occur fall			
Slippery condition of floor or non-slippery	*n* = 1383	*n* = 12,878	0.030
Slippery	92 (6.7)	1073 (8.3)	
Type of floor, concrete or others	*n* = 1382	*n* = 12,891	0.336
Concrete	1153 (83.4)	10,871 (84.3)	
Slope of floor, sloping or flat	*n* = 1388	*n* = 12,914	0.000
Sloping	260 (18.7)	1721 (13.3)	
Existing of obstacle, yes or none	*n* = 1388	*n* = 12,918	0.000
Yes	221 (15.9)	1487 (11.5)	

The significant main factors associated with severe injury in older adults patients with head injury after ground-level fall were: males (odds ratio 1.626, 95 % confidence interval 1.414–1.869, *p* = 0.000) aged 70 years or greater; time of injury, 00:00–05:59 rather than 18:00–23:59 (1.252, 1.028–1.524, *p* = 0.025); residential facility rather than transportation area (1.721, 1.391–2.128, *p* = 0.000); indoor rather than outdoor area (1.258, 1.046–1.514, *p* = 0.015); daily activity rather than unpaid activity (1.543, 1.075–2.212, *p* = 0.018); fall from stairs (1.835, 1.372–2.453, *p* = 0.000) or slip down on the same level rather than fall over (1.547, 1.321–1.812, *p* = 0.000); non-slippery floor rather than slippery conditions (1.399, 1.093–1.792, *p* = 0.008); and presence of obstacles on the floor (1.298, 1.061–1.588, *p* = 0.011) (Table 4).

**Table 4:** General characteristics of fall-related head injury by severity in the older adults

	Odds Ratio	95 % Confidence Interval	*P*
Univariate logistic regression analysis			
Age group, years old			
60 ~ 69	1.0		
70 ~ 79	1.362	1.200-1.547	0.000
≥ 80	1.331	1.153–1.536	0.000
Male, sex versus female	1.395	1.247–1.560	0.000
Season of injury occurrence			
Winter	1.0		
Spring	0.946	0.811–1.105	0.486
Summer	0.968	0.829–1.130	0.679
Autumn	0.916	0.789–1.063	0.247
Time of injury occurrence			
00:00–05:59	1.0		
06:00–11:59	0.923	0.768–1.109	0.392
12:00–17:59	0.913	0.777–1.074	0.272
18:00–23:59	0.780	0.662–0.919	0.003
Location of injury occurrence			
Residential facility	1.0		
Medical facility	0.923	0.654–1.303	0.650
Sports facility or work place	1.028	0.721–1.468	0.877
Transportation area	0.648	0.571–0.736	0.000
Public or commercial facility	0.887	0.733–1.074	0.219
Other outdoor area	0.904	0.660–1.238	0.528
Outdoor fall injury versus indoor fall injury	0.866	0.776–0.967	0.010
Activity during injury occurrence			
Daily activity	1.0		
Paid work	1.247	0.875–1.778	0.222
Unpaid work	0.793	0.579–1.087	0.149
Sports or leisure activity	0.819	0.703–0.954	0.010
Alcohol ingestion	0.825	0.708–0.961	0.014
Mechanism of fall			
Others including fall over	1.0		
Fall from stairs	1.889	1.623–2.198	0.000
Slip down on same level	1.339	1.183–1.517	0.000
Non-slippery, condition of floor versus slippery	1.275	1.023–1.590	0.031
Concrete, type of floor versus others	0.936	0.806–1.086	0.383
Sloping floor versus flat	1.499	1.298–1.731	0.000
Existing of obstacles on floor	1.456	1.248–1.697	0.000
Multivariate logistic regression analysis			
Age group, versus 60 ~ 69			
70 ~ 79	1.344	1.155–1.563	0.000
≥ 80	1.310	1.096-1.565-	0.003
Male, sex versus female	1.626	1.414–1.869	0.000
18:00–23:59, time of injury occurrence versus 00:00–05:59	0.799	0.656–0.973	0.025
Transportation area, location of injury occurrence versus residential facility	0.581	0.470–0.719	0.000
Indoor fall injury versus outdoor fall injury	1.258	1.046–1.514	0.015
Unpaid work, activity during injury occurrence versus daily activity	0.648	0.452–0.930	0.018
Mechanism of fall, versus others including fall over			
Fall from stairs	1.835	1.372–2.453	0.000
Slip down on same level	1.547	1.321–1.812	0.000
Non-slippery, condition of floor versus slippery	1.399	1.093–1.792	0.008
Existing of obstacles on floor	1.298	1.061–1.588	0.011

## Discussion

In this study, men were more likely than women to present to the ED with head injuries and men in the head injury group were also more likely than women to have suffered severe injuries. The overall incidence of fall-related injuries in the older adults is higher in women than in men, [5] but the mortality rate associated with such injuries was higher in men than in women [10, 12, 19]. In a previous study, after a fall injury, men had more head, face, and chest injuries than women, and women had more extremity injuries than men;[7] however, another study showed that the rate of head impact was more than twice as high in women than in men [24]. The difference in sex-specific mortality is likely attributable to the increased life expectancy of women vs. men [27] as well as sex-based differences in muscle strength. The higher mortality rate in older adults men after fall injuries may be related to several factors, such as their increased incidence of outdoor falls, their comparatively rapid decay of leg muscles coupled with higher overall activity levels, [20] their increased rates of comorbidity, and their greater tendency to participate in active behaviors, all of which would be expected to result in more severe injuries than those seen in women [10]. Other studies have shown that older women are at greater risk of indoor fall injuries because their leg muscles are weaker than those of men,[23] and that the ground-level fall injury risk is higher in women owing to an increased possibility of a loss of balance [28].

In this study, age was not an independent risk factor for head injury after ground-level fall in the older adults; however, those over the age of 69 in the head injury group were at greater risk of severe injury. In a previous study, patients over the age of 70 spent longer in the hospital and ICU and had an increased mortality rate than their younger counterparts [19]. Another study showed that the mortality rate according to age after a ground-level fall injury is sex-specific. The odds ratio of in-hospital mortality of the group older than 60 years vs. under 60 was higher in men, but it was not a significant risk factor in women aged 60–79 compared to those under 60 [21].

Alcohol use is a risk factor for fall injury in the older adults, possibly because blood alcohol levels after imbibing tend to remain higher in older people than young people owing to a decline in metabolic rate with age [29]. In this study, alcohol ingestion was a risk for head injury after ground-level falls in the older adults, but it did not affect the rate of severe injury in the head injury group. The prevalence of alcohol ingestion at the time of fall injury was higher in this study than in a previous study [18]. The definition of severe injury, which involved an emergency operation or admission to the ICU in this study, or the particular characteristics of the study subjects, all of whom presented to the university hospital ED, might affect this result. A head injury was more likely to result from a ground-level fall when alcohol was ingested than when no alcohol was ingested [12, 13]. Alcohol ingestion at the time of a ground-level fall tended to increase the rate of head injury among older people; also, men who suffered a ground-level fall were more likely to ingest alcohol than women and the rate of alcohol ingestion decreased with increasing age [18]. Alcohol impairs self-protective reflexes and makes it harder for an older adults to use their arms to control a fall, thus increasing the likelihood of head injury [13].

Environmental factors related to the floor are also important in ground-level fall injury in the older adults. In this study, the risk of head injury after a ground-level fall was higher if the floor was not slippery, made of concrete, sloped, and had obstacles and the risk of severe injury in the head injury group was higher when the floor was non-slippery and had obstacles. Although the floor environment did not affect injury severity, in men, the risk of severe injury was higher for outdoor falls from stairs [14]. Previous studies have shown that the characteristics of the floor and flooring materials affect the severity of fall injury among the older adults [11, 30, 31]. Fall injury increases when there are obstacles on the floor [17] and decreases when the floor is flat or soft [15, 30]. The risk of severe injury is higher on a non-slippery floor [14]. This may be related to an increase in outdoor falls, as the activity levels of older adults individuals tend to increase outdoors [15]. In addition, in this study, the risk of head injury was greater when the mechanism of injury was a fall from stairs compared to falling over, and the risk of severe injury in the head injury group was also increased. In previous studies, among those who suffered a fall injury outdoors, falls from stairs rather than non-head injury mechanisms were associated with a higher risk of severe injury due to the height difference [14, 16].

Although the degree of cognitive impairment, degree of independence in daily life, and degree of visual impairment may affect the incidence and severity of falls in the older adults, [24] those factors were not included in this study. Also, individual medical history or use of medications that could affect fall injury in the older adults were not investigated in this study. The subjects of this study may have suffered more severe injuries than those in a population-based study because all of the hospitals in this study were university training hospitals. Functional cognitive outcomes were not included in this study; moreover, severity was defined by clinical outcomes at the ED, not by objective data such as injury severity scale or Glasgow coma scale scores. These factors may have influenced the results. Although many previous studies defined the older adults as those aged 65 years or older, this study set the cut-off point for the older adults at 60 years old. Although this study has limitations related to descriptive analysis, environmental factors (such as slipperiness, slope, flooring material, and the presence of obstacles) that could influence ground-level fall-related injuries in the older adults were included in the analysis. The results of this study are expected to be utilised to help identify and improve upon environmental risk factors in order to reduce the risk of falls in the older adults.

Male sex, outdoor fall, alcohol ingestion, sloping floor, and presence of obstacles on the floor were risk factors associated with head injury after a ground-level fall in the older adults in this study. The severe head injury subgroup were more likely to be older males, to have suffered an indoor fall, and to have encountered obstacles on the floor. It is necessary to develop preventive programs, taking both individual and environmental characteristics into account, to reduce the socioeconomic costs associated with ground fall injury in the older adults in an aging era.

## Data Availability

The Korea Center for Disease Control and Prevention Agency (KDCA) has the authority to access and use this data. Data can be requested through the department of injury prevention of the KDCA by E-mail (kcdcinjury@korea.kr) or website (http://www.kdca.go.kr/).

## Abbreviations

TBI: Traumatic brain injury
ED: Emergency department
KDCA: Korea Center for Disease Control and Prevention Agency
ICD-10: International Statistical Classification of Diseases and Related Health Problems 10th Revision
EMR-ISS: Excess mortality ratio-adjusted injury severity score
ICU: Intensive care unit

## References

[1] Miyoshi Y, Kondo Y, Hirano Y, Ishihara T, Sueyoshi K, Okamoto K, Tanaka H (2020). Characteristics, injuries, and clinical outcomes of geriatric trauma patients in Japan: an analysis of the nationwide trauma registry database. Scientific reports.

[2] Deandrea S, Lucenteforte E, Bravi F, Foschi R, La Vecchia C, Negri E (2010). Risk factors for falls in community-dwelling older people: a systematic review and meta-analysis. Epidemiology.

[3] Harvey LA, Close JC (2012). Traumatic brain injury in older adults: characteristics, causes and consequences. Injury.

[4] Korhonen N, Niemi S, Parkkari J, Sievänen H, Kannus P (2013). Incidence of fall-related traumatic brain injuries among older Finnish adults between 1970 and 2011. Jama.

[5] O’Neill S, Brady RR, Kerssens JJ, Parks RW (2012). Mortality associated with traumatic injuries in the elderly: a population based study. Arch Gerontol Geriatr.

[6] Peel NM, Kassulke DJ, McClure RJ (2002). Population based study of hospitalised fall related injuries in older people. Inj Prev.

[7] Stevens JA, Rudd RA (2014). Circumstances and contributing causes of fall deaths among persons aged 65 and older: United States, 2010. J Am Geriatr Soc.

[8] Lozano R, Naghavi M, Foreman K, Lim S, Shibuya K, Aboyans V, Abraham J, Adair T, Aggarwal R, Ahn SY (2012). Global and regional mortality from 235 causes of death for 20 age groups in 1990 and 2010: a systematic analysis for the Global Burden of Disease Study 2010. Lancet.

[9] Liu SW, Obermeyer Z, Chang Y, Shankar KN (2015). Frequency of ED revisits and death among older adults after a fall. Am J Emerg Med.

[10] Alamgir H, Muazzam S, Nasrullah M (2012). Unintentional falls mortality among elderly in the United States: time for action. Injury.

[11] Bhattacharya B, Maung A, Schuster K, Davis KA (2016). The older they are the harder they fall: Injury patterns and outcomes by age after ground level falls. Injury.

[12] Chatha H, Sammy I, Hickey M, Sattout A, Hollingsworth J (2018). Falling down a flight of stairs: The impact of age and intoxication on injury pattern and severity. Trauma.

[13] Johnston JJ, McGovern SJ (2004). Alcohol related falls: an interesting pattern of injuries. Emerg Med J.

[14] Jung HY, Kim SH, Lee SC, Kim S, Cho GC, Kim MJ, Lee JS, Han C (2018). Relating factors to severe injury from outdoor falls in older people. Geriatr Gerontol Int.

[15] Kelsey JL, Procter-Gray E, Hannan MT, Li W (2012). Heterogeneity of falls among older adults: implications for public health prevention. Am J Public Health.

[16] Kim SH (2016). Risk factors for severe injury following indoor and outdoor falls in geriatric patients. Arch Gerontol Geriatr.

[17] Lim YM, Sung MH (2012). Home environmental and health-related factors among home fallers and recurrent fallers in community dwelling older Korean women. Int J Nurs Pract.

[18] Shakya I, Bergen G, Haddad YK, Kakara R, Moreland BL (2020). Fall-related emergency department visits involving alcohol among older adults. J Safety Res.

[19] Spaniolas K, Cheng JD, Gestring ML, Sangosanya A, Stassen NA, Bankey PE (2010). Ground level falls are associated with significant mortality in elderly patients. J Trauma.

[20] Stevens JA, Sogolow ED (2005). Gender differences for non-fatal unintentional fall related injuries among older adults. Inj Prev.

[21] Taira T, Morita S, Umebachi R, Miura N, Icimura A, Inoue S, Nakagawa Y, Inokuchi S (2015). Risk factors for ground-level falls differ by sex. Am J Emerg Med.

[22] Verma SK, Willetts JL, Corns HL, Marucci-Wellman HR, Lombardi DA, Courtney TK (2016). Falls and Fall-Related Injuries among Community-Dwelling Adults in the United States. PLoS One.

[23] Wolfson L, Whipple R, Derby CA, Amerman P, Nashner L (1994). Gender differences in the balance of healthy elderly as demonstrated by dynamic posturography. J Gerontol.

[24] Yang Y, Mackey DC, Liu-Ambrose T, Leung PM, Feldman F, Robinovitch SN (2017). Clinical Risk Factors for Head Impact During Falls in Older Adults: A Prospective Cohort Study in Long-Term Care. J Head Trauma Rehabil.

[25] Kim J, Shin SD, Im TH, Kug Jong L, Ko SB, Park JO, Ahn KO, Song KJ: Development and validation of the Excess Mortality Ratio-adjusted Injury Severity Score Using the International Classification of Diseases 10th Edition. Academic emergency medicine: official journal of the Society for Academic Emergency Medicine 2009, 16(5):454–464. 10.1111/j.1553-2712.2009.00412.x 19388920

[26] Lee H, Kim SH, Lee SC, Kim S, Cho GC, Kim MJ, Lee JS, Han C (2018). Severe Injuries from Low-height Falls in the Elderly Population. J Korean Med Sci.

[27] Life expectancy at birth [https://data.oecd.org/healthstat/life-expectancy-at-birth.html]

[28] Caserotti P, Aagaard P, Simonsen EB, Puggaard L (2001). Contraction-specific differences in maximal muscle power during stretch-shortening cycle movements in elderly males and females. Eur J Appl Physiol.

[29] Barry KL, Blow FC (2016). Drinking Over the Lifespan: Focus on Older Adults. Alcohol Res.

[30] Drahota AK, Ward D, Udell JE, Soilemezi D, Ogollah R, Higgins B, Dean TP, Severs M (2013). Pilot cluster randomised controlled trial of flooring to reduce injuries from falls in wards for older people. Age Ageing.

[31] Gustavsson J, Bonander C, Andersson R, Nilson F (2015). Investigating the fall-injury reducing effect of impact absorbing flooring among female nursing home residents: initial results. Inj Prev.

